# Prolongation of Pregnancy in Patients with HELLP Syndrome Using Methylprednisolone: A Retrospective Multicentric Analysis

**DOI:** 10.3390/life13041013

**Published:** 2023-04-14

**Authors:** Anna Katharina Hosten, Jennifer Bonitz, Volker Thäle, Michael Tchirikov

**Affiliations:** 1 Department of Obstetrics and Prenatal Medicine, Martin-Luther-University of Halle-Wittenberg, 06120 Halle (Saale), Germany; 2 Department of Paediatrics, Heinrich-Braun-Klinikum Zwickau, 08060 Zwickau, Germany; 3 Department of Gynecology and Obstetrics, Helios Hospital Sangerhausen, 06526 Sangerhausen, Germany

**Keywords:** HELLP syndrome, methylprednisolone, corticosteroids, pregnancy, prolongation, preterm birth, preeclampsia

## Abstract

Background: Immediate delivery is an established concept for preventing life-threatening complications in mothers with HELLP syndrome; however, it is associated with preterm births. Methods: Cases of HELLP syndrome diagnosed at the university hospitals of Halle and Magdeburg (Germany) were analyzed retrospectively. Each patient of the treatment group was administered 64 mg of methylprednisolone (MP) intravenously for 10 days, with the dosage being reduced by 50% every other day in patients from Halle (n = 65). Almost immediate delivery was performed in the control groups (n = 45, Halle; n = 28, Magdeburg). Results: Pregnancies in the treatment group were prolonged by 4 days (median 1–55 days). The platelet counts increased from 76,060 ± 22,900/μL to 117,430 ± 39,065/μL in the MP group compared with an increase from 66,500 ± 25,852/μL to 83,430 ± 34,608/μL in control group 1 and from 78,890 ± 19,100/μL to 131,080 ± 50,900/µL in control group 2 (*p* < 0.001). Severe neonatal complications were significantly reduced in the treatment group (*p* < 0.05): sepsis, 9.25% vs. 24%; ventilation, 44.6% vs. 46.5%; and infant death, 1.6% vs. 8.6%. Conclusions: In a selected collective of patients with HELLP syndrome, prolongation of pregnancy using MP treatment improved maternal and neonatal outcomes.

## 1. Introduction

HELLP syndrome, as a severe manifestation of pre-eclampsia, can significantly endanger the lives of mothers and fetuses [1,2].

HELLP syndrome refers to the development of hemolysis, elevated liver enzymes and low platelets in pregnant women. As the mother is in great danger of hemorrhaging due to low platelets, the fetus might be compromised by iatrogenic preterm birth to save the mother’s life. Immediate delivery is, therefore, often considered a remedy in this situation, as the symptoms of HELLP syndrome commonly improve after delivery. While HELLP syndrome mainly affects the mother, neonates can also be compromised. Neonatal morbidity and mortality do not primarily depend on the degree of the symptoms of the mother but rather on the degree of prematurity. While immediate delivery may be advantageous for the mother, neonates can be severely endangered if they are born extremely prematurely. 

HELLP syndrome usually develops between 27 and 37 weeks of gestation [3]. It rarely occurs before 27 gestational weeks and in certain cases it only manifests postpartum. Treatment options other than immediate delivery include administration of corticosteroids or plasma exchange. At the university hospital of Halle (Germany), we sometimes use intravenous application of steroids to improve the mother’s platelet count.

Magann et al. conducted a study on the use of antepartum corticosteroids as disease stabilizers in patients with HELLP syndrome and since then, discussion on how to handle HELLP syndrome as a severe complication of pregnancy has become more controversial. Prolongation of pregnancy to reduce fetal underdevelopment versus immediate delivery to reduce the mother’s symptoms after diagnosis with HELLP syndrome now seems possible [4].

Woudstra et al. reviewed the use of corticosteroids to treat HELLP syndrome in pregnancy [1]. The authors conclude that administration of corticosteroids, particularly dexamethasone, improves platelet counts; however, there is no clear evidence of improvement in clinical outcome. At the university hospital of Halle (Germany), methylprednisolone (MP) is the first-line treatment for HELLP syndrome. There are differences in corticosteroid use in pregnancy, depending on whether the target is the mother, the fetus or both. In HELLP syndrome—contrary to fetal lung maturation—the mother is the primary recipient of corticosteroids if HELLP symptoms need to be controlled. Corticosteroids used primarily to treat the pregnant mother should have a low ability to cross the placenta or be used in lower doses [5]. Betamethasone and Dexamethasone cross the placenta rather easily [6] and are therefore used to induce fetal lung maturation. In contrast, physiological cortisol has a lower ability to cross the placenta as it is rapidly inactivated by 11β-hydroxysteroid dehydrogenase type 2 [7,8]. 

In this retrospective study, we examined whether there was an improvement in the outcomes of mothers and children with HELLP syndrome following administration of corticosteroids.

## 2. Materials and Methods

Published studies on treatment of HELLP syndrome using corticosteroids is limited. Ethical considerations make randomized controlled trials difficult. In German law, the treatment of severe cases of HELLP syndrome using corticosteroids is possible in the form of so called ‘individual healing attempts’. We conducted a retrospective analysis of these cases to broaden the database. The treatment of patients in this study was conducted before the established guideline started to be implemented.

We analyzed 138 pregnant women with HELLP syndrome who were inpatients at the university hospitals of Halle and Magdeburg between 1990 and 2013. An internal review board vote was not necessary for this retrospective analysis.

Women with HELLP syndrome were identified by searching through birth books and documentation on the course of pregnancy, delivery, childbed and newborn period (extracted from the hospital’s archives, patient’s files and electronic databases). Patients from the university hospital of Magdeburg were also identified from patients’ files or electronic databases.

There was no clear date when treatment with MP was started, resulting in an overlap of therapies for HELLP syndrome. The study presented here analyzed cases from the university clinics of Halle and Magdeburg—the university clinics in the political unit of Sachsen-Anhalt/Germany. We included 110 women with a prepartum diagnosis of HELLP syndrome who were treated at the university hospital of Halle between 1990 and 2011 as well as 28 patients treated at the university hospital of Magdeburg between 2007 and 2013. The latter formed one of the control groups, as patients in Magdeburg did not receive steroids as a treatment for HELLP syndrome during this period. Overall, 138 patients were included in the analysis. Of those, 65 patients diagnosed in 1999–2011 received MP for prolongation of pregnancy (treatment group). Corticosteroids as standard therapy were introduced in Halle in 2001. However, five patients with pregnancies occurring after 2000 (in 2001, 2005 and 2008) were not treated with MP. Two precursor cases from 1999 and 2000 were treated with MP and were included in the treatment group.

A control group of 45 patients with HELLP syndrome diagnosed at the university hospital of Halle between 1990 and 2000 (plus five patients diagnosed in 2001, 2005 and 2008) was not treated with MP (control group 1). 

A second control group (control group 2) of 28 patients with HELLP syndrome who were treated at the university hospital of Magdeburg between 2007 and 2013 met the inclusion criteria. 

In control group 1 and 2, rapid delivery was sought following diagnosis of complete HELLP syndrome. The control groups were not treated with corticosteroids.

### 2.1. Pregnancy Prolongation

Provided that maternal and fetal parameters were stable, prolongation of pregnancy in the treatment group was possible. Patients were started on MP after diagnosis with HELLP syndrome. We commenced treatment with a 64 mg dosage and reduced this by 50% every other day. Methylprednisolone treatment ended after 10 days. Another cycle of 10 days of MP treatment was given if required due to an exacerbation in symptoms associated with HELLP syndrome. As there were no guidelines for MP treatment in our institution at that time, four patients were treated with different doses (3 × 80 mg, 4 × 60 mg, 128/96/64/32 mg). If delivery was necessary before reaching the tenth day of treatment, treatment was resumed after delivery and continued until a total of ten days of treatment was completed. If HELLP syndrome symptoms manifested only after delivery, then the ten-day MP treatment was administered after delivery.

### 2.2. Inclusion Criteria for Prolongation

An attempt at pregnancy prolongation was made for each patient in the treatment group who had not reached 32 weeks of gestation if maternal and fetal parameters were stable.

### 2.3. Exclusion Criteria for Prolongation

Concerning the maternal condition, indications for immediate delivery included neurological symptoms (headache, dizziness and changes in visual acuity as a sign of imminent preeclampsia), non-treatable hypertonus or kidney insufficiency, acute pulmonary edema, persistent right upper quadrant pain, persistent nausea and vomiting, progressive contractions, placental abruption and suspected cerebral hemorrhage or liver hematoma.

Concerning laboratory parameters, signs of DIC (parameters: Quick, INR, fibrinogen, D-dimers and progressive decline in platelet counts) were an exclusion criterion for prolongation.

Imminent fetal asphyxia, severe intrauterine growth retardation (IUGR), anhydramnios and intrauterine fetal death excluded prolongation of the pregnancy.

A prolongation of at least one day was possible in 35 of the 65 patients in the treatment group.

### 2.4. Maternal Complications

These included rupture of the liver capsule, eclampsia, preterm placental abruption, disseminated intravascular coagulation, pulmonary edema/pleural effusion, postpartum hemorrhaging, severe anemia with need of a transfusion of blood cell concentrates, ascites and maternal mortality.

### 2.5. Fetal Baseline Variables

Fetal baseline variables comprised birth weight, postpartum fetal condition (APGAR), respiratory stimulation measurements (mask, CPAP, intubation) and acid-base state as signs of intrauterine hypoxia with subsequent acidosis. Acidosis was defined as an umbilical cord pH of less than 7.2.

### 2.6. Monitoring the Fetal Condition

We monitored the fetuses to detect IUGR or placental insufficiency using cardiotocography controls, sonographic fetometry and Doppler sonography of the uterine and umbilical arteries.

We examined the following complications during the neonatal period: retinopathy (ROP), sepsis, cerebral hemorrhage (IVH), respiratory distress syndrome (IRDS), bronchopulmonary dysplasia and intrauterine fetal death. We also examined parameters such as length of ventilation and hospital stay (neonatal intensive care unit).

### 2.7. Statistical Analysis

Statistical analyses were conducted using Microsoft Excel^®^ and IBM SPSS Statistics 19.0^®^. Two independent samples were compared.

The arithmetic means and standard deviation were used to describe normally distributed variables, while the median and range of the data were calculated to describe skewed variables. Categorical variables were documented as case numbers and percentages. Confirmatory tests depended on the measured level of the target variable. The non-parametric tests used were chi-square test for nominal variables and binary logistic regression in the multifactorial model. Mann–Whitney U test was used to analyze variables that were not normally distributed. The t-test with Bonferroni correction was used to compare mean values of normally distributed data. Kaplan–Meier survival curve was used for graphical representation of time to a defined endpoint, and the corresponding curves were compared using log-rank test.

The level of significance was set at α = 0.05. For multiple comparisons, we used Bonferroni corrected thresholds like α = 0.05/2 = 0.025, for example for treatment group vs. control group 1.

## 3. Results

From January 1990 to December 2013, 146 pregnancies complicated by HELLP syndrome were documented at the university hospitals of Halle and Magdeburg. We included 138 of these patients in our study and divided them into three groups (65 vs. 45 vs. 28 patients).

Gestational ages in the different groups at time of diagnosis and time of delivery are given in Table 1. Diagnosis in the study group was made two weeks earlier than in the control groups (Table 1).

### 3.1. Signs and Symptoms

Table 2 compares the typical signs and symptoms associated with HELLP syndrome in the three groups. 

Majority of the pregnant women displayed typical signs of preeclampsia during or preceding HELLP symptoms. For example, hypertension with median blood pressure of 159.46 ± 18 systolic (systolic max. = 210 mmHg) over 96.08 ± 13.21 diastolic (diastolic max. = 130 mmHg). A total of 47 patients (43.1%) developed edema, while 40 patients (36.7%) suffered from proteinuria.

Significant differences were observed in the Doppler ultrasound findings; the study group seemed to be more severely compromised. Bias can be expected, although it is not possible to determine in which direction this bias occurred from our data due to the retrospective nature of the study.

A total of 25 patients (38.5%) vs. 24 (53.3%) presented with pathological cardiotocography at admission or later during their stay in hospital.

Documentation of the symptoms in control group 2 (Magdeburg) was not complete, as shown in Table 2.

### 3.2. Prolongation Data

**Figure 1:**
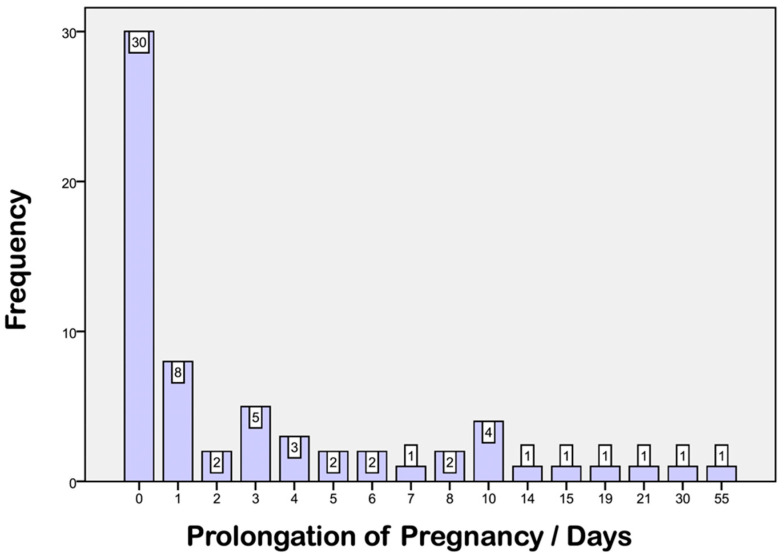
Frequency of prolongation time (days/frequency) in the treatment group.

A prolongation of pregnancy of at least one day was achieved in 35 patients in the treatment group (n = 65; 54%; Figure 1). A median of four days of prolongation was achieved (variation: 1–55 days). Maximum time of prolongation was 55 days in one case (less than 27 completed weeks of gestation at admission). No prolongation was achieved in 30 patients due to severely worsening fetal or maternal state, which necessitated delivery in less than 24 h. Treatment with MP was continued for each woman in the treatment group after delivery.

### 3.3. Changes in Laboratory Parameters

Slight improvements in laboratory parameters associated with HELLP syndrome were observed within a few days in the treatment group (due to treatment with MP) and in the control groups (due to immediate delivery) (Table 3).

An increase in platelets from 76,060 ± 22,900/μL (17,000–105,000/μL, vs. control group 1: 66,500 ± 25,852/μL, 21,000–105,000/μL vs. control group 2: 78,890 ± 19,100/μL) to 117,430 ± 39,065/μL (*p* = 0.0001) (control group 1: 83,430 ± 34,608/μL, *p* = 0.013; control group 2: 131,080 ± 50,900, *p* = 0.0001) was observed within the first three days of treatment with MP. The mean difference in platelets between day 1 and day 3 was 38,500/μL vs. 18,500/μL (*p* = 0.023) vs. 53,154/μL (*p* = 0.194).

**Figure 2:**
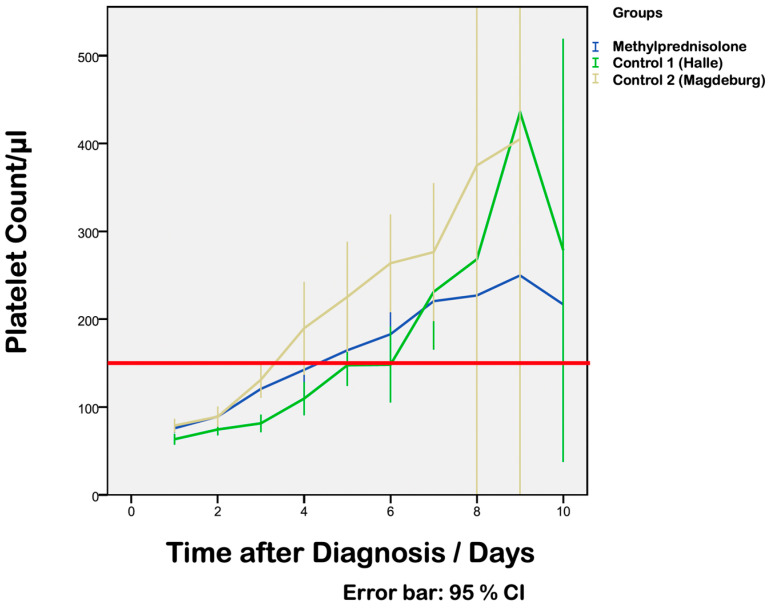
Median pre- and postpartum platelet counts in the treatment group following HELLP syndrome diagnosis and postpartum platelet counts in the control groups (blue: treatment group with MP; green: control group 1 from Halle; yellow: control group 2 from Magdeburg). The red line indicates the lower limit of the normal range of platelet counts. The x axis shows time after diagnosis in days and the y axis shows platelet counts per µL.

More than half of the patients in the treatment group (57.8%, n = 38) reached a platelet count within normal range 4 days after starting MP treatment (vs. control group 2: 42.9%, n = 12 vs. control group 1: 27.3%, n = 12; Figure 2).

Analysis of other laboratory values showed a decrease in aspartate transaminase (AST) levels in the treatment group within the first three days, from an initial 264 ± 318 U/L (variation: 30–2070 U/L, vs. control group 1; 240 ± 198 U/L, variation: 30–762 U/L vs. control group 2; 282 ± 300 U/L) to 72 ± 60 U/L (*p* = 0.0001) (control group 1: 126 ± 168 U/L; * *p* = 0.007, control group 2: 108 ± 78 U/L, *p* = 0.007). On day five of treatment with MP, 49 patients in the treatment group (43.9%) had AST levels less than 34.8 U/L; however, this was achieved in only 31.8% (n = 14) of patients in control group 1 and 14.3% of patients in control group 2 (*p* = 0.006). On average, normalization of values in the treatment group was reached after 6.2 days (95% CI = 336–408) vs. 6.8 days (95% CI = 367.8–444.6) and 7.3 days (95% CI = 390–480) in the control groups.

The initial level of LDH in the treatment group (694.2 ± 420 U/L, variation: 139.2–1840.2) was significantly lower than in control group 1 (1363.8 ± 1111.8 U/L, variation: 300–5772; *p* = 0.001). There was a decrease within the first three days to 205.8 U/L in the treatment group vs. 706.2 U/L (*p*= 0.121) in control group 1. Both groups still showed levels above the normal range at dismissal. LDH levels were not measured in control group 2.

Hemolysis can be detected by monitoring the level of haptoglobin. Comparisons between the different groups was difficult because haptoglobin was not, or only rarely, measured in the control groups. There was an increase from 16.91 ± 20 g/L (variation: <6–80) (n = 56) to 45 ± 29 g/L (n = 25) (*p* = 0.0001) within the first three days. This represents approximately three-fold increase. Both treatment and control groups showed a slow increase in haptoglobin levels. However, the levels in the control group fluctuated around significantly lower values over the entire observation period, indicating somewhat higher levels of hemolysis. 

### 3.4. Complications

**Figure 3:**
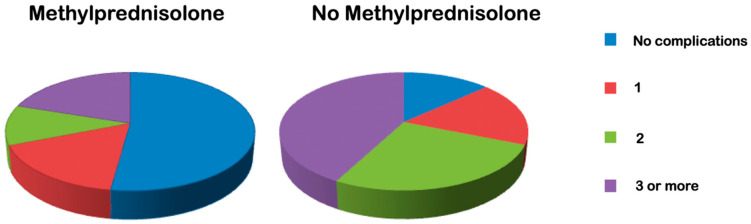
Frequency of peripartum complications in the treatment group (MP, **left**) vs. control group 1 (no MP, **right**).

Analysis of peripartum complications showed that more than half of the patients in the treatment group (52.3%, n = 34) underwent a peripartum course with no complications (vs. control group 1: only 13% with no peripartum complications; * *p* = 0.0001). On average, at least two complications occurred in the control group in contrast to the treatment group (Figure 3).

Study group and control group 1 had the greatest differences in the percentages of patients with anemia requiring transfusion. In the treatment group, only 19 patients (29.2%) developed anemia: in 14 cases (21.5%), red blood cell concentrates were needed, while platelet concentrates were needed in six cases (9.2%). In control group 1, 29 patients (64.4%; *p* < 0.0001) had anemia: 27 (53.3%) needed red blood cell concentrates, while 16 cases (35.6%) required platelet concentrates (*p* = 0.001) (Table 4).

There were no cases of intrauterine fetal deaths in the treatment group, whereas two fetuses in control group 1 died intrauterine (4.4%). 

A total of 14 (21.5%, treatment group) vs. 15 (33.3%, control group 1) vs. 6 (21.4%, control group 2) cases of pre- and postpartum bleeding complications occurred (Table 4).

No patient in any of the groups suffered a prepartum eclamptic seizure; however, two patients in the treatment group and one patient in control group 1 had a postpartum seizure.

There were significant differences in gestational age at the time of birth between the groups. This made comparisons of fetal outcomes difficult. Therefore, we divided the newborns into smaller groups. Due to the small number of cases in the individual groups, no true significant statements could be made initially. However, children in the treatment group—in cases where pregnancy prolongation was possible—were born with a higher mean birth weight. In particular, high birth weights were noted in cases were HELLP syndrome was diagnosed before 29 weeks of gestation and where a mean prolongation of 10 days was reached by administering MP. A multifactorial regression analysis of the outcome birth weight showed that the prolongation of pregnancy had a significant influence on weight gain.

Analysis of complications arising during the neonatal period showed that infants in the treatment group had a 65% lower relative risk (OR 0.355) of invasive ventilation via a tube (73% relative risk reduction, OR 0.27) at delivery, regardless of gestational age (Table 5). Analysis of the remaining complications, including retinopathy, cerebral hemorrhage, BPD and growth retardation showed no significant differences in the overall collective. 

## 4. Discussion

### 4.1. Definition of HELLP Syndrome

HELLP syndrome is defined based on a combination of several laboratory parameters during pregnancy: hemolysis (haptoglobin deficiency), elevated liver enzymes (elevated AST, ALT) and low platelet counts (less than 100,000/µL) [9]. The acronym HELLP was first introduced by Weinstein in 1982 as a variant of preeclampsia following collection and review of a larger number of case reports [10]. However, its management remains controversial up to this day. Fully developed HELLP syndrome bears severe risks for mother and child and can be life-threatening for the mother. Therefore, for a long time, immediate delivery to save the mother seemed to be the correct approach. Besides prompt delivery, maternal stabilization, control of severe hypertension, administration of antenatal corticosteroids between 24 and 34 weeks of gestation and magnesium sulfate therapy are part of the current treatments [3]. Neonates may be at a high risk of premature birth and subsequent sequelae depending on the weeks of gestation at diagnosis of HELLP syndrome. Based on the concept that HELLP syndrome is a type of severe inflammatory preeclampsia, treatment with ani-inflammatory drugs or immunosuppressants has been considered. Martin et al. recommended aggressive use of potent corticosteroids as a cornerstone for the management of HELLP syndrome [11]. 

### 4.2. Guidelines

The 2019 German guidelines on Hypertension in Pregnancy state that there is no evidence-based use of corticosteroids in the treatment of HELLP syndrome and preeclampsia. Therefore, corticosteroids are not recommended for the treatment of HELLP syndrome [9]. This recommendation is based on the NICE guidelines on hypertension in pregnancy, which advise against the use of dexamethasone and betamethasone in the treatment of HELLP syndrome. It states that corticosteroids have only one advantage when used in the treatment of HELLP syndrome: improving platelet count [12]. This point of view was shared by a report of the American College of Obstetricians and Gynecologist’s Task Force on Hypertension in Pregnancy, which showed evidence of improved platelet counts in randomized trials of corticosteroid treatment [13]. 

Additionally, the German guidelines advise against postpartum treatment of preeclampsia or HELLP syndrome using corticosteroids [9].

### 4.3. Study Approach

This retrospective study examined whether the materno-fetal outcomes of HELLP syndrome can be improved by administering MP. Additionally, the occurrence of hemolysis and elevations in the levels of liver enzymes following steroid use was monitored. 

Pregnancies of patients in the treatment group were prolonged with MP; however, patients in control group 1 did not receive MP and their pregnancies were not prolonged. Patients in control group 2 met the inclusion criteria for prolongation but were treated only by near immediate delivery and not with corticosteroids. Corticosteroids were administered in some cases to induce lung maturation.

### 4.4. Comparing Results of this Study with Those from the Literature 

Typical symptoms such as right upper quadrant pain, headache and other symptoms of preeclampsia did not differ between the groups. The results were consistent with data from the study by Rath et al. [14]. 

Analysis of cardiotocography results showed a tendency towards more pathological findings in the control group and there were fewer pathological cardiotocography findings in the treatment group (38.5% vs. 53.3%, *p*= 0.126, s. Table 2). This may be an effect of the corticosteroids. However, the conditions of the mothers and children in control group 1 were rather unstable and patients in control group 2 could not be analyzed due to missing Doppler sonography and cardiotocography data.

**Figure 4:**
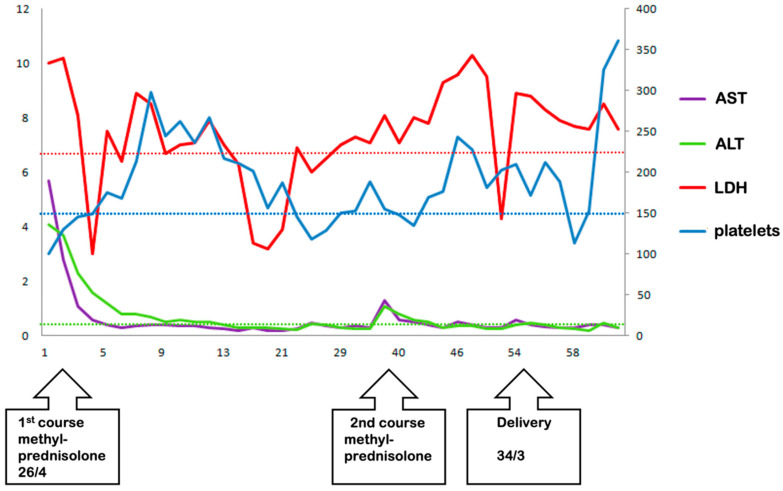
Liver enzymes and platelet count in a pregnancy where maximum prolongation was achieved. AST and ALT levels returned to normal after the first course of MP. LDH levels fell below the lower limit of the normal range; however, the effect was less pronounced compared with that of AST and ALT levels. Platelet counts remained well above the lower limit of the normal range during most of the observation period. The total duration of prolongation of this pregnancy was 55 days.

The first three days of treatment with MP showed significant improvements in all laboratory results (platelet count, AST, ALT, LDH and haptoglobin) (Figure 4). Slow improvements in laboratory parameters were detected in the control groups, but only after delivery. There was a more rapid increase in platelet counts in the treatment group compared with control group 1 (6.3 vs. 4.7 days, *p* = 0.0001).

In 1999, Fischer et al. studied 58 patients with HELLP syndrome. They aimed to prolong pregnancies where HELLP syndrome was diagnosed before 32 weeks of gestation and to stabilize the maternal condition in patients in whom HELLP syndrome was diagnosed after 32 gestational weeks. The prospectively studied treatment group consisted of 38 patients at less than 32 weeks gestation who received 40 mg MP i.v. per day. A median pregnancy prolongation of 6.5 days was reached. Pregnancies at less than 32 gestational weeks (n = 16) were prolonged for a median of 12 days [2]. Our retrospective observational study found a median additional time of four days. Pregnancies at less than 34 gestational weeks were prolonged by 6 days, while those at less than 29 weeks of gestation were prolonged by 10 days. These results are comparable to those of the study by Fischer et al. 

The increase in platelet counts was similar in the treatment group and ranged from 69 800 ± 23 000/µL (s.d) (vs. 61 900 ± 32 900/µL in the control group) to 231 700 ± 104 000/µL (*p* < 0.01) [2]. The higher absolute platelet counts in the study by Fischer et al. might be due to the longer observation period of 10 days compared with our results, which were recorded within the first three days of treatment. Another difference between our observations and those of the study by Fischer et al. was the dosage of MP: Fischer et al. administered 40 mg MP everyday while we started with a dosage of 64 mg and reduced this by half every other day.

We also discovered some cases of rebound thrombocytosis in the control group (n = 16, 35.5%) from day nine onwards. The median platelet count was 563 Gpt/L (410–911 Gpt/L), with the increase being an additional risk factor. Only 24% of patients in the treatment group developed thrombocytosis with lower median platelet count (476 Gpt/L, 402–826 Gpt/L) vs. control group 2 (more than 400 Gpt/L, maximum 606 Gpt/L, n = 4). We assumed a protective effect of MP against rebound thrombocytosis as the median platelet counts in the treatment group were lower. A case report from 1989 showed that thrombocytosis is a rare but sometimes fatal risk factor for HELLP syndrome: a 27-year-old woman died from cerebral infarction secondary to carotid artery thrombosis 9 days after cesarean delivery. Her platelet count at admission on day nine was 601,000/mm^3^. Her lowest platelet count, measured on the third postoperative day, was 29,000/mm^3^ [15]. The maximum platelet count in our control group was close to that of the patient in the case report described above. Unfortunately, the literature on rebound thrombocytosis in patients with HELLP syndrome is limited. It is unknown whether the lack of case reports is due to a lack of thrombocytosis in patients with HELLP syndrome or whether it is because no one has described it.

Analysis of other laboratory values in patients whose pregnancies were prolonged in the study by Fischer et al. showed decreased AST levels, from 129 ± 143 U/L (52–428; vs. control 130 ± 114 U/L) to 14 ± 4.6 U/L (*p*< 0.001) [2].

The patients in Fischer’s study also had comparable decreases in LDH levels, from 548 ± 353 U/L (226–1441 vs. control 666 ± 202 U/L) to 263 ± 94 U/L (*p* < 0.01) [2].

The changes in hemoglobin levels in our study do not differ significantly from those in the study by Fischer et al. who found approximately five-fold increase, from 17.0 ± 12.1 mg/dL (range < 10–48 vs. control 12.5 ± 8.6 mg/dL) to 91.5 ± 12.5 (*p* < 0.01).

### 4.5. Is Immediate Delivery Still Justified?

Neonatal morbidity and mortality does not depend primarily on the maternal symptoms of HELLP syndrome but on the degree of preterm births [16,17]. 

Based on the observed stabilization of laboratory results, immediate delivery following diagnosis of HELLP syndrome does not seem justified anymore. The most important effect of treatment with MP was a prolongation of pregnancy and, therefore, additional time for the fetus to grow intrauterine.

Abramovici et al. presented a study of 269 neonates who were delivered preterm due to HELLP syndrome, partial HELLP syndrome or severe preeclampsia. The number of complications in mothers with HELLP syndrome and preeclampsia were not significantly different. Fewer neonatal morbidity and mortality cases were noted at more advanced stages of pregnancy compared with earlier stages of pregnancy. The authors concluded that in severe preeclampsia, neonatal morbidity and death are associated with gestational age rather than with the presence or absence of HELLP syndrome [18]. Based on these findings, we aimed to prolong pregnancy in patients with HELLP syndrome using MP treatment. Prolongation of pregnancy past 34 gestational weeks was not sought because maternal risks were higher than neonatal risks resulting from preterm birth. We found that the effect of prolongation of pregnancy was represented by better 1-minute-APGAR. Infants in the treatment group had higher median birth weights compared with infants in the control group. These beneficial effects were the result of a few more days of prolongation of pregnancies at lower gestational weeks. Additionally, the umbilical cord blood was less acidic in neonates in the treatment group compared with neonates in the control group. This difference was strongest in pregnancies at 32 weeks of gestation, with a difference of 0.14 (arterial) and 0.11 (venous). Neonates in the treatment group were more stable at delivery compared with those in the control group. While the APGAR is observer-dependent, umbilical cord acidity is objectively measured based on the pH. 

### 4.6. Limitations of the Study

There are several limitations in our observations, mainly due to the retrospective nature of the study. The control group from Magdeburg differed in several ways from the patients in Halle, including in terms of diagnosis and treatment. There is also potential bias due to the process of selecting patients with HELLP syndrome who were eligible for prolongation of pregnancy at the two university hospitals.

Concerns have been raised over the effects of comparatively low doses of corticosteroids on fetal lung maturation. The long-term development of children treated with steroids in utero is an important topic for future studies. A systematic review did not exclude the risk of cognitive impairment following lung maturation [19].

In summary, our data show that treating HELLP syndrome using MP can significantly prolong pregnancy by improving the condition of the mother. This, in turn, improves the fetal outcome as well.

## 5. Conclusions

The results of the present study are of interest in the routine use of MP for prolongation of pregnancy. This holds true in perinatal centers with the possibility of optimal monitoring of maternal and fetal conditions and the prerequisite of immediate readiness for C-section in case of sudden complications.

The results indicate that MP therapy not only improves the clinical and laboratory outcomes of mother and child, it also reduces the number of postpartum complications. For example, fewer red blood cells and platelet concentrates have to be transfused [20]. The therapy also reduces the risk of complications from blood transfusion. From an economic point of view, MP therapy is clearly superior to transfusion or alternative HELLP syndrome therapies such as plasmapheresis. Plasmapheresis may give results similar to MP therapy; however, it is more invasive, dangerous and, above all, expensive [21].

Multicentric studies with significant patient sizes are needed to confirm these results and, if necessary, to further optimize the therapy or identify the potential limits of this therapy. Additionally, the question of optimal dosage and duration of MP administration needs to be addressed. 

Finally, a closer examination of cases of rebound thrombocytosis, including its causes, consequences and treatment options, might be of interest.

## Figures and Tables

**Table 1:** Gestational ages (and level of significance) at diagnosis and delivery (study group versus control groups 1 and 2).

Gestational Week	Study Group n = 65	Control Group 1 n = 45	*p*	Control Group 2 n = 28	*p*
at diagnosis	31^2^ ± 4 wks	33^3^ ± 3 wks	0.011	33^0^ ± 4 wks	0.06
at delivery	31^6^ ± 4 wks	33^4^ ± 3 wks	0.029	33^1^ ± 4 wks	0.16

**Table 2:** Symptoms associated with HELLP syndrome in the three groups (incomplete documentation in group 2).

	Study Group n = 65 (%)	Control Group 1 n = 45 (%)	*p*	Control Group 2 n = 28 (%) *
Epigastric Pain	56 (86.2)	38 (84.4)	0.803	19 (68)
Nausea/Vomiting	34 (52.3)	21 (46.7)	0.561	8 (29)
Severe Headache	16 (24.5)	17 (37.8)	0.139	2 (7.1)
Flickering	9 (13.8)	8 (17.8)	0.575	1 (3.6)
Hypertension	47 (72.3)	39 (86.7)	0.073	21 (79)
Edema	24 (36.9)	23 (52.3)	0.112	-
Proteinuria	21 (32.3)	19 (43.2)	0.278	-
Increased Resistance in Uterine Artery	35 (53.8)	9 (20.0)	0.0001	-
Notching	29 (44.6)	9 (20)	0.008	-
Increased Resistance in Umbilical Artery	29 (44.6)	7 (15.6)	0.001	-
Cardiotocography Path	25 (38.5)	24 (53.3)	0.126	4 (14.3)

* Selected data from control group 2 are shown.

**Table 3:** Difference in the improvements in laboratory values in the first five days of commencing treatment (study group) and after delivery (control groups 1 and 2).

Difference	Group	Day 1 to 3	*p*	Day 1 to 4	*p*	Day 1 to 5	*p*
Platelet count	Study group	38.500	-	75.647	-	94.977	-
	Control 1	18.757	0.023	45.545	0.114	80.241	0.490
	Control 2	53.150	0.194	110.14	0.260	151.25	0.122
AST	Study group	3.294	-	3.479	-	2.975	-
	Control 1	1.806	0.157	2.916	0.624	3.182	0.836
	Control 2	3.280	0.700	3.450	0.988	3.800	0.620
ALT	Study group	1.841	-	2.022	-	1.487	-
	Control 1	1.012	0.173	1.796	0.784	1.584	0.885
	Control 2	0.900	0.314	1.07	0.47	1.600	0.899
LDH	Study group	3.431	-	4.318	-	5.165	-
	Control 1	11.77	0.121	12.46	0.749	16.28	0.082

**Table 4:** Maternal complications (comparison of treatment group with control groups 1 and 2).

Type of Complication	Study Group n = 65 (%)	Control Group 1 n = 45 (%)	*p*	Control Group 2 n = 28 (%)	*p*
Premature detachment of the placenta	1 (1.5)	6 (6.7)	0.16	-	-
Anemia	19 (29.2)	29 (64.4)	<0.001	12 (43)	0.2
Transfusion (erythrocytes)	14 (21.5)	27 (53.3)	0.001	5 (18)	0.7
Transfusion (thrombocytes)	6 (9.2)	16 (35.6)	0.001	3 (11)	0.8
Triple I	11 (16.9)	14 (31.1)	0.08	8 (28.6)	0.2
Rupture of the liver capsule	1 (1.5)	1 (2.2)	0.79	-	-
Hemorrhaging	14 (21.5)	15 (33.3)	0.17	6 (21.4)	0.9
Respiratory problems	3 (4.6)	3 (6.7)	0.64	3 (11)	0.27
Eclamptic seizure	2 (3.1)	1 (2.2)	0.79	-	-

**Table 5:** Complications arising during the neonatal period (comparison of treatment and control groups).

Type of Complication	Study Group (n = 65)	Control Group (n = 71)	*p*	Odds Ratio	95% CI
Mechanical Ventilation	29 (44.6%)	33 (46.5%)	0.028	0.355	0.14–0.90
Intubation	14 (21.5%)	19 (26.8%)	0.014	0.270	0.1–0.77
Sepsis	6 (9.25%)	17 (24%)	0.001	0.111	0.03–0.39
Infant Death	1 (1.6%)	6 (8.6%)	0.046	0.101	0.011–0.96
Cerebral hemorrhage	8 (12.5%)	5 (7%)	0.965	0.971	0.26–3.5
Retinopathy	13 (20.3%)	5 (7.4%)	0.532	1.553	0.39–6.17
BPD	11 (17.2%)	4 (6%)	0.363	1.844	0.49–6.89
SGA	35 (53.8%)	28 (39.4%)	0.340	1.418	0.69–2.9

## Data Availability

Not applicable.
